# Do sex differences in paediatric type 1 diabetes care exist? A systematic review

**DOI:** 10.1007/s00125-022-05866-4

**Published:** 2023-01-26

**Authors:** Silvia A. G. de Vries, Carianne L. Verheugt, Dick Mul, Max Nieuwdorp, Theo C. J. Sas

**Affiliations:** 1 Department of Vascular Medicine, Amsterdam University Medical Centers, Amsterdam, the Netherlands; 2 Diabeter, Center for Paediatric and Adult Diabetes Care and Research, Rotterdam, the Netherlands; 3 Department of Paediatrics, Division of Paediatric Endocrinology, Erasmus University Medical Center, Sophia Children’s Hospital, Rotterdam, the Netherlands

**Keywords:** Child, Diabetes mellitus, Narrative synthesis, Sex differences, Systematic review, Type 1 diabetes

## Abstract

**Aims/hypothesis:**

Sex differences are present in cardiovascular care and in outcomes among adults with type 1 diabetes mellitus, which typically commences in childhood. Whether sex influences care and outcomes in childhood is not known. This systematic review provides an overview of sex differences in children with type 1 diabetes, focusing on patient and disease characteristics, treatment, comorbidities and complications.

**Methods:**

Literature in MEDLINE up to 15 June 2021 was searched, using the terms diabetes mellitus, sex characteristics, sex distribution, children and/or adolescents. All primary outcome studies on children with type 1 diabetes that mentioned a sex difference in outcome were included, with the exception of qualitative studies, case reports or case series. Studies not pertaining to the regular clinical care process and on incidence or prevalence only were excluded. Articles reporting sex differences were identified and assessed on quality and risk of bias using Joanna Briggs Institute critical appraisal tools. Narrative synthesis and an adapted Harvest plot were used to summarise evidence by category.

**Results:**

A total of 8640 articles were identified, rendering 90 studies for review (*n*=643,217 individuals). Studies were of observational design and comprised cohort, cross-sectional and case–control studies. Most of the included studies showed a higher HbA_1c_ in young female children both at diagnosis (seven studies, *n*=22,089) and during treatment (20 out of 21 studies, *n*=144,613), as well as a steeper HbA_1c_ increase over time. Many studies observed a higher BMI (all ages, ten studies, *n*=89,700; adolescence, seven studies, *n*=33,153), a higher prevalence of being overweight or obese, and a higher prevalence of dyslipidaemia among the female sex. Hypoglycaemia and partial remission occurred more often in male participants, and ketoacidosis (at diagnosis, eight studies, *n*=3561) and hospitalisation was more often seen in female participants. Most of the findings showed that female participants used pump therapy more frequently (six studies, *n*=211,324) and needed higher insulin doses than male participants. Several comorbidities, such as thyroid disease and coeliac disease, appeared to be more common in female participants. All studies reported lower quality of life in female participants (15 studies, *n*=8722). Because the aim of this study was to identify sex differences, studies with neutral outcomes or minor differences may have been under-targeted. The observational designs of the included studies also limit conclusions on the causality between sex and clinical outcomes.

**Conclusions/interpretation:**

Sex disparities were observed throughout diabetes care in children with type 1 diabetes. Several outcomes appear worse in young female children, especially during adolescence. Focus on the cause and treatment of these differences may provide opportunities for better outcomes.

**Registration:**

This systematic review is registered in PROSPERO (CRD42020213640)

**Graphical abstract:**

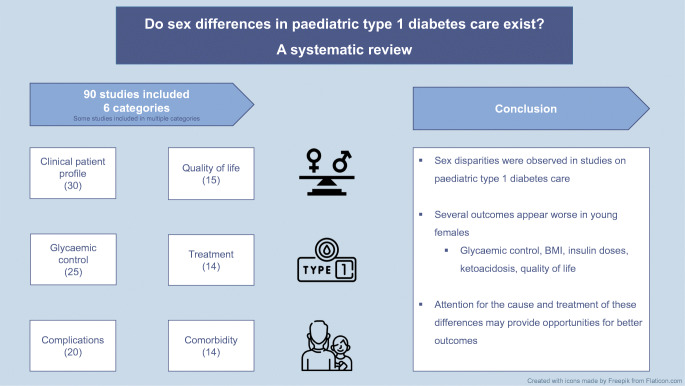

**Supplementary Information:**

The online version contains peer-reviewed but unedited supplementary material available at 10.1007/s00125-022-05866-4.

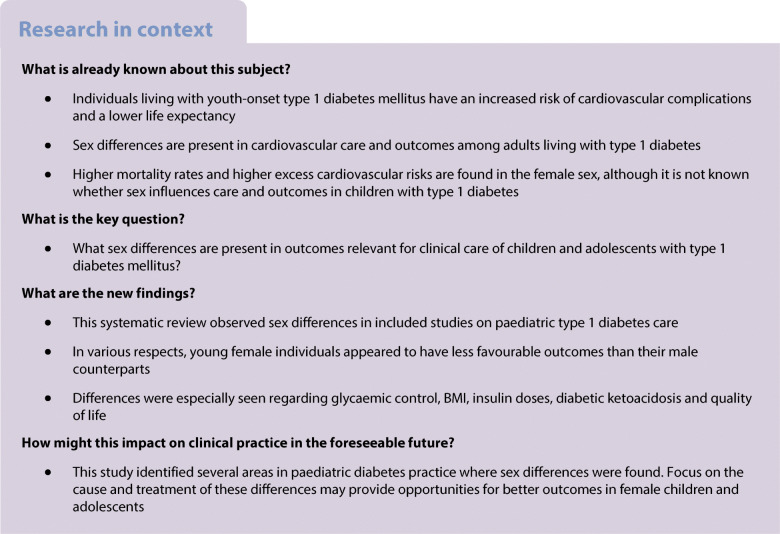



## Introduction

Diabetes mellitus is one of the major causes of morbidity and increased mortality rates worldwide [1, 2]. Global estimates show that more than a million children and adolescents are affected by type 1 diabetes [1, 2]. Many of these individuals experience microvascular and macrovascular complications later in life. Moreover, individuals with youth-onset type 1 diabetes mellitus have a lower life expectancy, despite many national and international initiatives to turn this tide [3–5]. This warrants a better understanding and identification of possible factors of influence in the young diabetes population. Among adults with diabetes, sex is recognised as a potent factor of influence, leading to increased attention for a tailored approach in men and women [6–8]. The importance of this approach is highlighted by the fact that female individuals with type 1 diabetes have an increased risk of all-cause mortality compared with male individuals, with vascular mortality being an important contributing force [8–10].

The excess cardiovascular risk in women has been associated with biological, pharmacological and care provision aspects [11]. Biological theories on body composition and differences in adipose tissue distribution, which influences the development of insulin resistance, are particularly applicable to type 2 diabetes mellitus. Yet sex differences among adults with type 1 diabetes are less well understood. Considering that sex differences are present in early life, and that complications and mortality risk increase with disease duration, skewed cardiovascular risk profiles may start to develop in childhood. Similar to adults, the influence of sex hormones on diabetes treatment and outcomes during puberty has been questioned [12, 13]. Moreover, metabolic control and treatment of diabetes in children and adolescents is influenced by a complex interaction between biological, psychological and social aspects, including sex [14]. Awareness of the role and extent of sex differences in paediatric diabetes practice is therefore important for optimal diabetes care and prevention of long-term complications. However, as yet, a comprehensive summary of all sex differences observed in paediatric diabetes care has not been provided.

By means of a systematic review, this study aims to give an overview of sex differences in children and adolescents with type 1 diabetes in daily paediatric care, with a focus on patient and disease characteristics, treatment, comorbidities and complications.

## Methods

### Literature search and study selection

A systematic literature search was performed in the MEDLINE database on 15 June 2021. Articles up until 14 June 2021 were included. In the title field, the following MESH terms and corresponding title and abstract free terms ([tiab]) were entered: diabetes mellitus AND (gender OR sex OR sex factors OR sex characteristics OR sex distribution) AND (pediatric OR paediatric OR pediatrics OR child OR youth OR adolescent). The terms did not include diabetes type in order to ensure the inclusion of studies that comprised more than one diagnosis type. Additionally, no term related to sex differences was added so that the literature could be assessed in a maximally objective fashion. The filter ‘humans’ was applied. For reasons of clarity, only titles in the English language were included. Titles and abstracts were screened on topic, and articles were included if they fit all three of the following criteria: (1) the study population included individuals with type 1 diabetes; (2) the study population included children or adolescents; and (3) a sex difference in outcome was described in either the title or abstract. All primary outcome articles were included with the exception of qualitative study designs, case reports and case series. Reviews, systematic reviews and meta-analyses were excluded. All selected abstracts were imported in Rayyan and assessed by two researchers on eligibility criteria (SAGdV and CLV) [15]. When abstracts did not provide sufficient information, full texts were screened. Titles were excluded for the following reasons: no full text was available; only patients >18 years old were part of the study population; no paediatric subanalysis was included; no sex differences were reported in the subpopulation with diabetes; no sex difference was described or found in the paediatric subpopulation; study participants were a highly selected subgroup such as a diabetes camp or a specific subpopulation; studies pertained to incidence or prevalence only; pre-clinical studies or genetic studies; insufficient information on sex analysis was provided; or the sex differences pertained to variables not used in the regular daily care process of children with type 1 diabetes. The selected studies that met all inclusion criteria were assessed on methodological quality and risk of bias using Joanna Briggs Institute (JBI) critical appraisal checklist tools for reporting as a guideline [16]. The tools used depended on the study design and included analytical cross-sectional studies, cohort studies and case–control studies. Most of the included cohort studies were observational and did not contain two groups, rendering the checklist item on similarity between groups not applicable. This item was adjusted by giving a clear description of the population and setting, as this was deemed particularly relevant for the study quality. Studies were excluded if the overall appraisal of the study was considered to be poor quality. Quality was considered poor if studies did not comply with all of the following criteria: the objective or the characteristics of the study population and setting were clearly defined; sufficient information on the outcomes was provided in the methods section; confounders were at least identified; and valid conclusions were drawn. Studies fulfilling all these criteria were considered either fair or good quality, and were included for data extraction. Distinction between good and fair quality was made based on the number of remaining questions on the checklists that could be answered affirmatively. The quality was considered good if the questions could all but one be answered with ‘yes’. If two or more items were considered unclear or negative, the quality of the study was considered fair. The findings were reported using the checklist in the PRISMA (preferred reporting items for systematic reviews and meta-analyses) statement [17]. The protocol of this review may be found in the PROSPERO International prospective register of systematic reviews (www.crd.york.ac.uk/PROSPERO; CRD42020213640).

### Data extraction and analysis

Selected studies were arranged in six categories: clinical patient profile; glycaemic control; comorbidity; complications; quality of life (QoL); and treatment. Clinical patient profile was divided into the following subcategories: duration of symptoms; remission; weight and BMI; BP; dyslipidaemia; antibodies; and C-peptide levels. Data was extracted from the included studies with a designed data extraction form including study characteristics, study design, country, the setting and year in which the study took place, sample characteristics, diabetes diagnosis and method, follow-up duration (when applicable), variables measured and outcomes related to sex differences. The large heterogeneity in study types, settings and outcomes rendered the extracted data unsuitable for meta-analysis. In addition to narrative synthesis, an adapted version of the Harvest plot was used to visually summarise the evidence [18]. The plots display all included studies by outcome category and combine information on study quality, study size and direction of outcomes. To illustrate the effect sizes within categories, relevant outcome measures for both female and male participants were reported in the outcome column of the plot. All applicable ORs or rates within the categories were selected, unless a different outcome measure in the study was deemed more informative, and display was regardless of significance of outcomes. The narrative synthesis summarises the included studies and highlights important findings or nuances in the extracted data. Final conclusions were based on the outcome direction of all studies within a category, corresponding to the results in the plots.

## Results

**Fig. 1:**
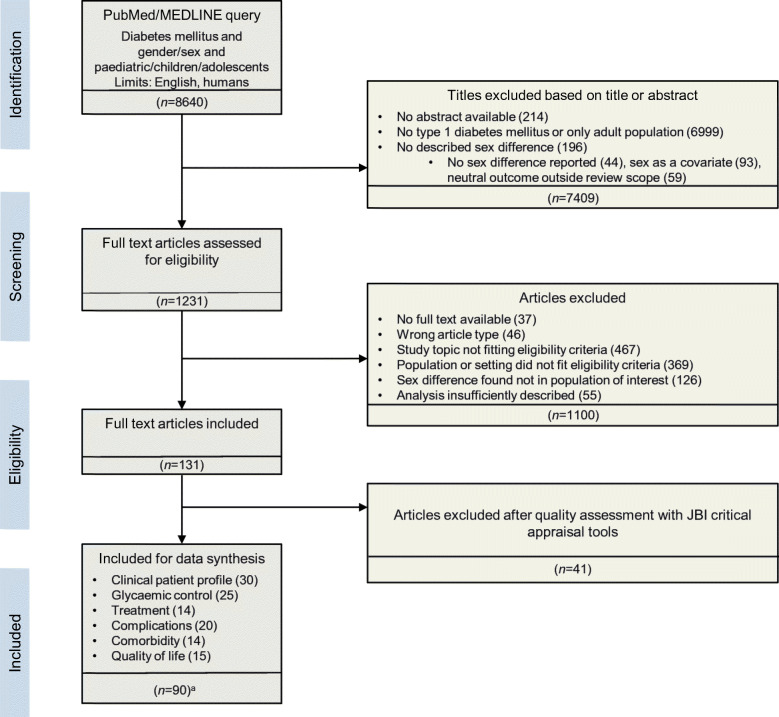
Flowchart on selection of articles included for synthesis. ^a^Several studies were included in more than one category. JBI, Joanna Briggs Institute

**Fig. 2:**
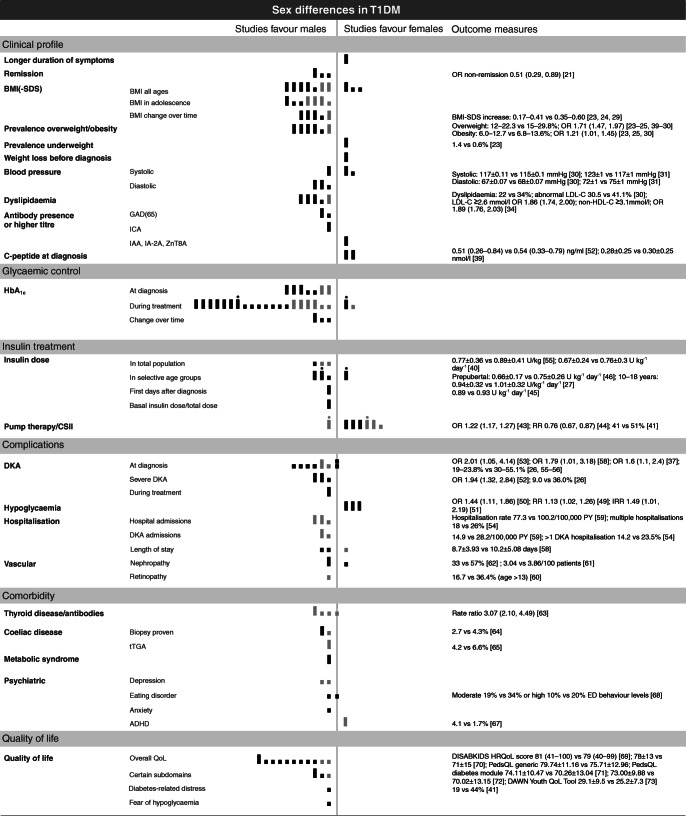
Overview of outcomes regarding sex differences in clinical profile, glycaemic control, insulin treatment, complications, comorbidity and QoL in children. The position of bars indicates whether the study outcome is favourable for male or female participants. Higher outcomes of complications or presence of comorbidity is scored as favourable for the other sex. Bar size reflects the number of participants (small, <1000; large, ≥1000). Bar colour reflects study quality (black, high quality; grey, fair quality). Outcome measures for male participants are listed first, followed by values for female participants (i.e. males vs females). Values are reported as means (95% CI), means ± SD, or medians with range. A circle above the bar indicates that the same study is scored on both outcome sides because of contrasting study results. For all references and sample sizes per study see electronic supplementary material (ESM) Tables 1, 2. ADHD, attention deficit hyperactivity disorder; ED, eating disorder; GAD(65), glutamic acid decarboxylase (65); HRQoL, health-related quality of life; IAA, insulin autoantibodies; IA-2A, islet antigen-2 autoantibodies; IRR, incidence rate ratio; LDL-C, LDL-cholesterol; non-HDL-C, non-HDL-cholesterol; PY, person-years; T1DM, type 1 diabetes mellitus; tTGA, tissue transglutaminase antibodies; ZnT8A, zinc transporter 8 antibodies

The literature search yielded 8640 studies, as illustrated in Fig. 1. These articles were screened on title and abstract, leaving 1231 articles that were assessed on the predefined eligibility criteria. This rendered 131 studies for quality assessment, 90 of which were suitable for synthesis (*n*=643,217 individuals). All included articles had an observational design and comprised cohort, cross-sectional and case–control studies. Data were often derived from national diabetes registries, medical chart reviews and databases registering details of the care process, and were regularly based on multicentre, international or European collaborations. Figure 2 provides an overview of all studies with outcomes on sex differences in type 1 diabetes (the subgroups are discussed below).

### Clinical profile

**Fig. 3:**
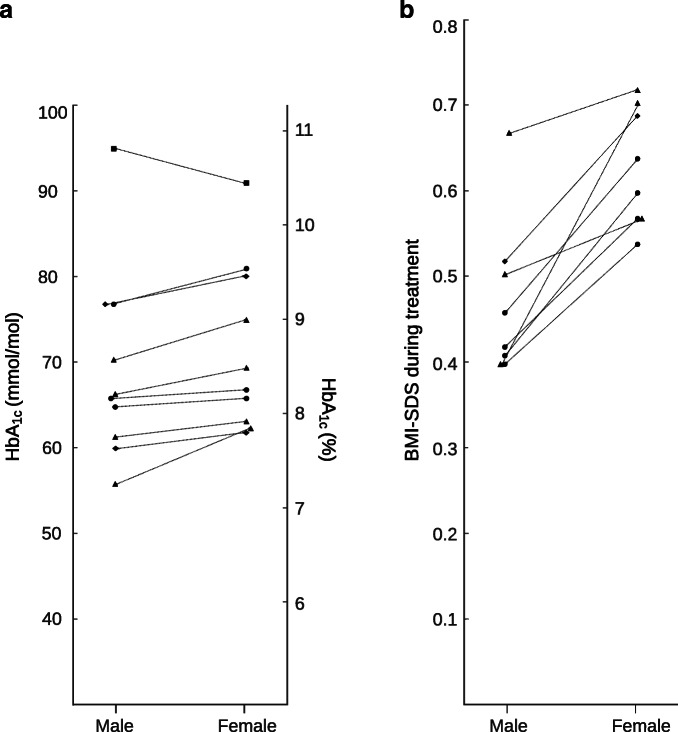
(**a**) Mean HbA_1c_ per study during follow-up for young male and female participants. Studies shown provided HbA_1c_ values stratified by sex (see ESM Table 3). Overall mean HbA_1c_ was calculated for subgroups. (**b**) BMI-SDS during treatment per study for young male and female participants. Studies provided BMI-SDS values stratified by sex (see ESM Table 4). Overall mean BMI-SDS was calculated for subgroups. Circles, children aged ≤18 years; triangles, adolescents aged 10–18 years; diamonds, outcomes significant in adolescence only; squares, young children aged 0–9 years (measured as GHb)

Figure 2 shows that more female participants than male participants displayed symptoms for longer than 4 weeks prior to diagnosis of type 1 diabetes [19]. As for antibodies, GAD autoantibodies or GAD65 antibodies seemed more common in female participants. The presence of islet cell antibodies (ICA) did not differ between the sexes but higher titres were seen in female participants. Positivity for ICA and GAD65 together was observed more often in female participants. Male participants more frequently had a positive outcome for insulin autoantibodies, islet antigen-2 autoantibodies and zinc transporter 8 antibodies [19]. Studies observed that male participants entered a partial clinical remission phase more often, had a longer remission phase and had a lower chance of non-remission (OR 0.51 [95% CI 0.29, 0.89]; *p*=0.018), especially those aged under 10 [20, 21] years. Older age at diabetes onset predicted a longer remission phase in male participants, while older age predicted shorter remission in female participants [22]. Differences related to physical examination were also found. As illustrated in Fig. 2 and Fig. 3b, BMI or BMI SD score (BMI-SDS) during treatment was almost invariably higher among female participants (all ages, seven out of ten studies [*n*=89,700]; adolescence, seven studies [*n*=33,153]), as was being overweight (22.3 vs 27.2%) or obese (12.7 vs 13.6%) [23–25]. In contrast, a lower BMI-SDS was seen in female participants at diagnosis or shortly thereafter (mean ± SD 0.56 ± 0.10 vs 1.04 ± 0.10; *p*<0.001), and relative weight loss before diagnosis was also higher in female participants than in male participants [19, 26]. Male participants were underweight during treatment more often than female participants (1.4 vs 0.6% prevalence) [23]. Furthermore, a distinction was seen between age categories. Four studies showed a larger increase in BMI(-SDS) over time in female participants and four different authors observed a higher BMI-SDS only during adolescent ages. Accordingly, studies that solely included adolescents showed results on BMI in disfavour of female participants, as was the case with age before transition (mean ± SD BMI-SDS 0.82 ± 0.67 for female participants vs 0.54 ± 0.87 for male participants; *p*=0.0008) [27, 28]. Additionally, BMI-SDS significantly increased by diabetes duration in female participants only (mean ± SD: <2 years 0.39 ± 0.05; 2–5 years 0.49 ± 0.04; and >5 years 0.60 ± 0.04; *p*<0.0001) and BMI-SDS increased more during insulin therapy (0.44 vs 0.18, *p*<0.0001) [23, 29]. Furthermore, one study on BP clinically observed a higher systolic BP (SBP) in male participants (mean ± SD 117 ± 0.11 vs 115 ± 0.1 mmHg; *p*<0.001) and a higher diastolic BP (DBP) in female participants (mean ± SD 68 ± 0.07 mmHg) when compared with male participants (mean ± SD 67 ± 0.07 mmHg; *p*<0.001) [30]. Another study also observed similar differences in SBP (mean ± SD: 123 ± 1 vs 117 ± 1 mmHg in male vs female participants; *p*<0.0001) and DBP (mean ± SD 72 ± 1 vs 75 ± 1 mmHg in male vs female participants; *p*<0.01) only for the age category 15–18 years [31]. Ambulatory 24 h BP measurement showed that female sex was strongly related with higher SBP and DBP [32]. Regarding laboratory measurements, the percentage of female participants with dyslipidaemia appeared to be higher compared with their male counterparts (five studies, *n*=50,827). These studies reported increased LDL-cholesterol, increased non-HDL levels, higher triglycerides, increased HDL-cholesterol and higher levels of total cholesterol in female participants [29, 30, 33–35]. The odds of having dyslipidaemic LDL-cholesterol (≥2.6 mmol/l; OR 1.86 [95% CI 1.74, 2.00]) and non-HDL-cholesterol (≥3.1mmol/l; OR 1.89 [95% CI 1.76, 2.03]) was higher for female participants [34].

### Glycaemic control

Outcomes on sex and glycaemic control were expressed as glycated haemoglobin (GHb) or HbA_1c_. Figure 2 shows that all studies showed higher HbA_1c_ outcomes for female participants, at diagnosis (seven studies, *n*=22,089), during treatment (20 out of 21 studies, *n*=144,613) and with regard to HbA_1c_ increase over time (three studies, *n*=8,536). In contrast, one study showed that young male participants had higher GHb at initial and follow-up measurements [36]. When stratified by HbA_1c_ levels, male participants had a lower risk of poor or moderate glycaemic control (RR reduction 0.5 [95% CI 0.2, 0.9]), and the percentages of female participants in the disadvantageous groups were higher [37, 38]. Some studies focused on specific age categories, or found significant differences in certain groups, as illustrated by Fig. 3a. At diabetes diagnosis, one study found higher HbA_1c_ levels in female participants of all ages but a significant difference was seen only for those aged 6–15 years old (mean ± SD: 6–10 years, 87.9 ± 21.2 [10.2%] vs 95.4 ± 24.4 mmol/mol [10.9%]; 11–15 years, 99.7 ± 24.8 [11.3%] vs 104.8 ± 27.3 mmol/mol [11.7%]) [39]. Another study showed a significant difference in HbA_1c_ between female and male participants during treatment only above the age of 12 years (mean ± SD 9.9 ± 2.0% [84.7 mmol/mol] vs 9.5 ± 2.0% [80.3 mmol/mol]; *p*<0.025) [40]. Other authors showed a higher HbA_1c_ during follow-up for female participants of all ages but this also became more obvious in the older age groups (mean ± SD: 10–14 years, 62 ± 12 mmol/mol [7.8%] vs 64 ± 12 mmol/mol [8.0%], *p*=0.01; 15–18 years, 65 ± 15 mmol/mol [8.1%] vs 68 ± 15 mmol/mol [8.4%], *p*<0.001) [39]. Additionally, four studies focused solely on glycaemic control in adolescent populations and showed a 0.22% (95% CI 0.08, 0.35; *p*<0.01) higher HbA_1c_ in female participants, and a mean HbA_1c_ of 56 ± 12.9 mmol/mol (7.3%) in female participants vs 62.4 ± 15.4 mmol/mol (7.9%) in male participants [27, 41]. Studies that reported age-adjusted HbA_1c_ levels reported higher values in female participants (mean ± SD: 8.20 ± 0.10% [66.1 mmol/mol] vs 8.06 ± 0.10% [64.6 mmol/mol]; *p*<0.0001) [23].

### Insulin treatment

The use of continuous subcutaneous insulin infusion (CSII) was highest in female participants in most of the study populations included (six studies, *n*=211,324), which is illustrated in Fig. 2 [42–44]. In the first days after diagnosis, a significantly higher administered mean insulin dose (ID) was reported in female vs male participants (0.89 vs 0.93 U kg^−1^ day^−1^) [45]. During treatment, studies reported a significantly higher mean daily ID in U/(kg body weight) in female participants. In prepubertal female individuals , a 14% higher daily ID was observed compared with male individuals [46], and in female participants aged 10–18 years the ID was 0.07 U/kg (95% CI 0.04, 0.09; *p*<0.0001) higher [27]. One study (indicated by the circle in Fig. 2) reported contrasting outcomes and showed that the daily ID was significantly higher for female participants aged 3–13 years old and male participants aged 14–18 years old [47]. A lower and more optimal ratio between the daily basal ID and the total ID was observed in male participants [48].

### Complications and comorbidity

Figure 2 shows three studies that reported a higher risk of severe hypoglycaemia in male participants (RR 1.13 [95% CI 1.02, 1.26]; *p*=0.02) [49–51]. In contrast, female participants appeared more likely to present with diabetic ketoacidosis (DKA) at diabetes diagnosis (eight studies, *n*=3561; OR 2.01 [95% CI 1.05, 4.14]; *p*=0.048) as well as during treatment (more than one DKA hospitalisation, 23.5% vs 14.2%; *p*<0.0001). The risk of severe DKA was also higher in female participants (36 vs 9.0%; *p*<0.05) [26, 37, 52–56]. The incidence of DKA increased with age in female participants, and markedly so in those aged >13 years [57]. Accordingly, hospitalisation rates (in general and related to DKA) were reported to be higher in female participants, and these participants had longer recovery times and length of hospital stay (mean ± SD: 10.2 ± 5.08 vs 8.7 ± 3.93 days; *p*=0.013) [58]. In contrast, a Canadian study observed prolonged hospitalisation in male participants [59]. Among the few studies reporting microvascular complications in children, the prevalence of retinopathy and nephropathy was higher in the female sex [60, 61]. One study found the non-albuminuric phenotype to occur more often in the male sex [62]. Studies also observed that antithyroid antibodies were more common in the female sex, as was hyperthyroidism and hypothyroidism, with hypothyroidism being more pronounced (rate ratio of female vs male sex 3.12 [95% CI 2.10, 4.63]) [63]. Individuals with concomitant coeliac disease more often appeared to be female, and this was confirmed by a higher prevalence of tissue transglutaminase antibodies and biopsy-proven coeliac disease among the female sex (4.3 vs 2.7%, *p*<0.001) [64, 65]. In a Brazilian group of adolescents with type 1 diabetes and the metabolic syndrome, the majority of the study population (75%) was female [66]. Among adolescent girls, higher rates of eating disorders, anxiety-related disorders and depression were found. One study observed that attention-deficit hyperactivity disorder was diagnosed two to three times more often in boys [67, 68].

### Quality of life

To assess QoL, different validated scales were used, such as the PedsQL, DISABKIDS and DQOL questionnaires, rendering studies with smaller sample sizes (15 studies, *n*=8722). Figure 2 demonstrates that all included studies reported lower QoL scores among the female sex [69–73]. In children of all ages, the difference in overall QoL and in the worries and health perceptions subscale became more pronounced in adolescence [27, 74]. One study found that parents of male children aged <8 years reported significantly higher diabetes-specific QoL than parents of female children [75]. Of note, nine out of 15 studies solely focused on adolescent ages (range 10–19 years old). Three of these studies found a lower QoL in female children only on subscales such as mental health, self-esteem, impact of diabetes, communication and worries. Moreover, female adolescents more often had moderate to severe diabetes-related distress (44% vs 19%) and scored higher on fear of hypoglycaemia [41].

## Discussion

This systematic review on sex differences in children with type 1 diabetes has shown that several outcomes appear to be worse in the female sex when compared with their male counterparts, particularly regarding BMI, glycaemic control, ID, diabetic ketoacidosis and QoL.

Multiple sex differences in the clinical profile of children with type 1 diabetes were observed. We found a higher HbA_1c_ at diagnosis and during treatment in female participants. In adults, studies have also shown that female participants with type 1 diabetes have worse or similar glycaemic control, despite more intensive treatment strategies to reach HbA_1c_ targets [76]. In line with this, higher IDs were observed in female participants. This suggests that it may be more difficult for female individuals to achieve the same level of glycaemic control. This sexual dimorphism may be caused by the biological influence of sex steroids, as oestrogen and androgens are known to have different effects on body composition and energy metabolism. Indeed, female hormonal spikes in puberty influence insulin sensitivity in type 1 diabetes [12, 13]. In female children without diabetes, reduced insulin sensitivity during puberty is compensated by increased endogenous secretion, whereas in type 1 diabetes the reduced sensitivity leads to a higher insulin need and might cause worse glycaemic control [77, 78]. Yet, a higher dose of prescribed exogenous insulin per kg of body weight might also be the result of worsening glycaemic control.

This review shows an unambiguously steeper BMI increase among female children, especially in adolescence. This is in line with results previously found in individuals with and without diabetes [79–81]. During puberty, male children develop more lean body mass whereas female children gain more fat mass, a physiological change which might be amplified in type 1 diabetes by the amount of insulin used [82]. Indeed, a relationship between insulin therapy intensity and BMI(-SDS) increase has been observed in previous studies [81, 83]. In the current study, the relationship between BMI-SDS increase and both diabetes duration and the amount of insulin used was stronger in the female sex, possibly suggesting that female children with type 1 diabetes are at higher risk of weight gain during puberty [23, 29]. One may speculate that the observed worse glycaemic control and higher IDs result in compensatory eating habits to avoid severe fluctuations in glucose levels and therefore lead to weight gain. Another explanation for the observed worse glycaemic control and higher BMI-SDS may be unhealthy behaviour habits other than overeating. Adding to this, other studies have previously shown that eating disorders are more prevalent among the female sex, and even more so in those living with type 1 diabetes [84, 85]. Finally, restriction of insulin as a weight-loss strategy has been suggested as a theory for higher HbA_1c_ in a selective group of female adolescents, although this contradicts the observation of overall higher BMI [86].

The observed disparities between sexes in BMI, ID and glycaemic control in our study populations can partially be attributed to puberty, yet this does not explain potential disparities in younger children. Factors that may play an important role at prepubertal ages are differences in the distribution of fat, insulin resistance, behavioural factors, growth hormone and the early influence of sex steroids [79, 87, 88]. Treatment bias to the disadvantage of young girls may also influence daily clinical care, potentially affecting the treatment of risk factors. In fact, studies in adults have observed differences in disfavour of women in relation to prescriptions and the achievement of target lipid levels and BP; our findings suggest that this disparity starts as early as adolescence [76].

Some findings are in line with the general population and are therefore not specific for children living with type 1 diabetes, such as female participants experiencing a lower QoL [89, 90]. Hormonal fluctuations during puberty and differences in coping mechanisms may potentially influence perceived QoL in adolescents [90]. Sex differences in disease-related behaviours and attitudes may play an additional role in young female individuals with diabetes [91]. These differences are especially relevant for clinical practice, as QoL can interfere with treatment adherence and glycaemic control. Increase in QoL may serve as an important tool towards further improvement of glycaemic control and reducing the risk of long-term complications [27].

Our findings suggest multiple outcomes less favourable in female children that create an unfortunate clinical profile, especially among those nearing young adulthood. It is concerning that many of the reported outcomes with a female sex bias are known contributors of a higher cardiovascular risk. Additionally, the outcomes of this study once again underline the importance of highlighting sex-specified outcomes in studies. Particular attention should be paid to sex differences within age categories by distinguishing children from adolescents, as they are clearly clinically different. Moreover, the findings of this review may guide towards more targeted studies on sex differences in the subdomains discussed. The unfavourable risk profile and related mortality risk in the female sex might start in the early years of the disease. This raises the question of whether young female children living with type 1 diabetes should be targeted more intensively on cardiovascular risk prevention, especially during or even before adolescence. Screening strategies and interventions that improve QoL and alleviate psychiatric comorbidities also seem warranted. Overall, in-depth evaluation of female individuals in clinical diabetes care and focusing on effective sex-specific interventions in the clinical research setting are necessary to create a future equally bright for both sexes.

This study is the first to provide a complete overview of the current literature on sex differences in paediatric type 1 diabetes care. It particularly focuses on disparities relevant for clinicians in daily diabetes care. The large number of studies reporting comparable outcomes in favour of one sex within several clinical categories renders the evidence increasingly convincing. An adapted version of the Harvest plot was used to synthesise the body of evidence relevant to this research question in a systematic way, emphasising quality of evidence and study size [18]. During the analyses, multiple measures were taken to ensure that only intermediate- or high-quality studies were included. As for limitations, the findings must be interpreted with caution because no meta-analysis was performed and because the studies included were of observational design, therefore the associations found do not automatically imply causality between sex and clinical outcomes. Despite efforts to be inclusive, narrative synthesis may have introduced selection bias in the results, yet we believe this to be limited by the representation of all studies in the Harvest plots. Additionally, the search was selectively performed in MEDLINE, potentially leading to some studies from other databases being missed. Moreover, the search strategy focused on sex differences, causing studies with a neutral outcome to be under-targeted with this query, possibly highlighting one side of the clinical picture or underreporting studies with a neutral or minor difference in sex and outcome. The influence of publication bias might be present as well, and sex differences that were not reported in an abstract might have been missed. Nonetheless, studies will generally mention the most remarkable findings in an abstract, so we believe the main body of evidence on sex differences to be covered by this systematic review.

In conclusion, sex disparities are observed in a variety of daily clinical variables and outcomes in the paediatric type 1 diabetes population, specifically among female adolescents. Most striking differences are seen regarding glycaemic control, BMI, ID, DKA and QoL. These differences suggest a less favourable clinical profile for young female individuals, with potential consequences later in life.

## Supplementary information

ESM Tables (PDF 420 kb)

## Data Availability

All data generated or analysed during this study are included in this article or its supplementary material files. Further enquiries can be directed to the corresponding author.

## Abbreviations

BMI-SDS: BMI SD score
CSII: Continuous subcutaneous insulin infusion
DBP: Diastolic BP
DKA: Diabetic ketoacidosis
GHb: Glycated haemoglobin
ICA: Islet cell antibodies
ID: Insulin dose
QoL: Quality of life
SBP: Systolic BP
