# Supplementary material for: Do sex differences in paediatric type 1 diabetes care exist? A systematic review
Source: Diabetologia. 2023 Jan 26;66(4):618–30. doi: 10.1007/s00125-022-05866-4 (PMC9947056; doi:10.1007/s00125-022-05866-4)
Supplement: Supplementary file 1 — (PDF 420 kb) [file 125_2022_5866_MOESM1_ESM.pdf]

Electronic Supplementary Material

**ESM Table 1** Details of studies included in the systematic review			
**Category**	**Studies favour males**	**Studies favour females**	**Outcome measures**
**Clinical profile**			
Longer duration of symptoms (n=4,993)		Turtinen 2018 (n=4,993) [1]	
Remission (n=6,476)	Dost 2007 (n=6,123) [2]<br>Marino 2017 (n=204) [3]<br>Ortqvist 1997 (n=149) [4]		OR non remission 0.51 (0.29-0.89) [3]
BMI all ages (n=89,700)	Maffeis 2018 (n=23,026) [5]<br>Plamper 2017 (n=1,294) [6]<br>Schwab 2010 (n=33,488) [7]<br>Dost 2008 (n=2,105) [8]<br>Łuczyński 2011 (n=500) [9]<br>Kapellen 2014 (n=25,762) [10]<br>Łuczyński 2014 (n=1,237) [11]	Islam 2014 (n=1,975) [12]<br>Kibirige 2003 (n=84) [13]<br>Cutfield 2011 (n=229) [14]	
BMI in adolescence (n=33,153)	BMI-SDS:<br>Manyanga 2016 (n=377) [15]<br>Birkebaek 2018 (n=11,025) [16]<br>Hanberger 2018 (n=14,383) [17]<br>Samuelsson 2016 (n=4,239) [18]<br><br>BMI:<br>Hoey 2001 (n=2,101) [19]<br>Dorchy 1997 (n=144) [20]<br>Mortensen 1988 (n=884) [21]		
BMI change over time (n=43,278)	Maffeis 2018 (n=23,026)[5]<br>Moore 2019 (n=7,002) [22]<br>Phelan 2020 (n=11,513) [23]<br>Łuczyński 2011 (n=500) [9]<br>Łuczyński 2014 (n=1,237) [11]		BMI-SDS increase:<br>0.17 vs. 0.35 [9]<br>0.41±0.05 vs. 0.60±0.04 (>5 years) [5]<br>0.18 vs. 0.44 [11]
Prevalence overweight/obesity (n=70,782)	Maffeis 2018 (n=23,026) [5]<br>Minges 2017 (n=5,529) [24]<br>Moore 2019 (n=7,002) [22]<br>Schwab 2010 (n=33,488) [7]<br>Łuczyński 2011 (n=500) [9]<br>Łuczyński 2014 (n=1,237) [11]		Overweight<br>12.0 - 22.3 vs. 15.0 - 29.8%:<br>- 18.9 vs. 27.19% [24]<br>- 22.3 vs. 27.2% [5]<br>- 12 vs. 15 % [7]<br>- 21.9 vs. 29.8% [9]<br>- 16.6 vs. 23.1% [11]<br>OR 1.71 (1.47-1.97) [24]<br><br>Obesity<br>6.0 - 12.7 vs. 6.8 - 13.6%:<br>- 12.7 vs. 13.6% [24]<br>- 7.3 vs. 6.8% [5]<br>- 6 vs. 8% [7]<br>OR 1.21 (1.01-1.45) [24]
Prevalence underweight (n=23,026)		Maffeis 2018 (n=23,026) [5]	1.4 vs. 0.6%[5]
Weight loss before diagnosis (n=4,993)		Turtinen 2018 (n=4,993) [1]	

Systolic blood pressure (n=36,540)	Dost 2008 (n=2,105) [8]	Schwab 2010 (n=33,488) [7]<br>Mortensen 1994 (n=947) [25]	117±0.11 vs. 115±0.1 mmHg [7]; 123±1 vs. 117±1 mmHg (15-18 years old) [25]
Diastolic blood pressure (n=36,540)	Dost 2008 (n=2,105) [8]<br>Schwab 2010 (n=33,488) [7]<br>Mortensen 1994 (n=947) [25]		67±0.07 vs. 68±0.07 mmHg [7]; 72±1 vs. 75±1 mmHg (15-18 years old) [25]
Dyslipidaemia (n=50,827)	Gomes 2013 (n=1,692) [26]<br>Kosteria 2019 (n=14,290) [27]<br>Schwab 2010 (n=33,488) [7]<br>Silverio 2019 (n=120) [28]<br>Łuczyński 2014 (n=1,237) [11]		Dyslipidaemia 22 vs 34% [7]; Abnormal LDL-C 30.5 vs 41.1% LDL-C ≥2.6 mmol/l OR 1.86 (1.74-2.00) Non-HDL-C ≥3.1mmol/l OR 1.89 (1.76-2.03) [27]
GAD65 antibodies (n=5,142)	Turtinen 2018 (n=4,993) [1]<br>Ortqvist 1997 (n=149) [4]		
ICA antibodies (n=4,993)	Turtinen 2018 (n=4,993) [1]		
IAA, IA-2A, ZnT8A antibodies (n=4,993)		Turtinen 2018 (n=4,993) [1]	
C-peptide at diagnosis (n=4,922)		Szypowska 2018 (n=1,098) [29]<br>Samuelsson 2013 (n=3,824) [30]	0.51[0.26-0.84] vs.0.54 [0.33-0.79] ng/ml [29]; 0.28±0.25 vs. 0.30±0.25 nmol/L [30]
**Glycaemic control**			
HbA_{1c} at diagnosis (n=22,089)	Samuelsson 2013 (n=3,824) [30]<br>Turtinen 2018 (n=4,993) [1]<br>Szypowska 2018 (n=1,098) [29]<br>Ortqvist 1997 (n=149) [4]<br>Quinn 2006 (n=247) [31]<br>Hanberger 2014 (n=7,539) [32]<br>Samuelsson 2016 (n=4,239) [18]		
HbA_{1c} during treatment (n=144,613)	Craig 2007 (n=2,312) [33]<br>Gomes 2013 (n=1,692) [26]<br>Hoey 2001 (n=2,101) [19]<br>Moore 2019 (n=7,002) [22]<br>*Plamper 2017 (n=1,294) [6]<br>Maffeis 2018 (n=23,026) [5]<br>Schwab 2010 (n=33,488) [7]<br>Cutfield 2011 (n=229) [14]<br>Khanolkar 2017 (n=364) [34]<br>Lawes 2014 (n=155) [35]<br>Ortqvist 1997 (n=149) [4]<br>Rohan 2014 (n=240) [36]<br>Springer 2006 (n=667) [37]<br>Forsander 2017 (n=453) [38]<br>Hanberger 2014 (n=7,539) [32]<br>Hanberger 2018 (n=14,383) [17]<br>Mcknight 2015 (n=44,058) [39]<br>Samuelsson 2016 (n=4,239) [18]<br>Mortensen 1988 (n=884) [21]<br>Yazar 2019 (n=61) [40]	*Plamper 2017 (n=1,294) [6]<br>Allen 1992 (n=277) [41]	*Contrasting result: Overall higher HbA_{1c} in females, higher in males 13.5-15 years old [6]
HbA_{1c} change over time (n=8,536)	Moore 2019 (n=7,002) [22]<br>Plamper 2017 (n=1,294) [6]<br>Rohan 2014 (n=240) [36]		
**Treatment**			
Insulin dose in total population (n=1,428)	Ortqvist 1997 (n=149) [4]<br>Demir 2015 (n=395) [42]<br>Mortensen 1998 (n=884) [43]		0.77±0.36 vs. 0.89±0.41 IU/kg [42] 0.67±0.24 vs. 0.76±0.3 U kg^{-1} day^{-1} [43]

Insulin dose in selective age group (n=24,626)	Hoey 2001 (n=2,101) [19]<br>*Wiegand 2008 (n=22,177) [44]<br>Komulainen 1998 (n=348) [45]	* Wiegand 2008 (n=22,177) [44]	10-18 years: 0.94±0.32 vs. 1.01±0.32 U kg^{-1} day^{-1} [19]<br>Prepubertal age: 0.66±0.17 vs. 0.75±0.26 IU kg^{-1} day^{-1} [45]<br>*Contrasting result: The ID was higher in males at 14–18 years old and higher in females at 3–13 years old. [44]
Insulin dose first days after diagnosis (n=1,680)	Herbst 2005 (n=1,680) [46]		0.89 vs. 0.93 IU kg^{-1} day^{-1} [46]
Basal insulin dose/total dose (n=19,687)	Rasmussen 2019 (n=19,687) [47]		
Pump therapy/CSII (n=211,324)	*Hanberger 2018 (n=14,383) [17]	Bächle 2013 (n=1,473) [48]<br>Sherr 2016 (n=54,410) [49]<br>van den Boom 2019 (n=96,547) [50]<br>Forsander 2017 (n=453) [38]<br>*Hanberger 2018 (n=14,383) [17]<br>Mcknight 2015 (n=44,058) [39]	OR 1.22 (1.17-1.27) [49]; RR 0.76 (0.67-0.87) [48]; 41 vs. 51% [38]<br>*Contrasting result: In Austria and Germany a higher proportion of girls used CSII, in Sweden males used CSII more often.

ESM Table 2 Details of studies included in the systematic review

**Category**	**Studies favour males**	**Neutral outcome**	**Studies favour females**	**Outcome measures**
**Complications**				
DKA at diagnosis (n=3,561)	Aminzadeh 2019 (n=297) [51]<br>Cutfield 2011 (n=229) [14],Khanolkar 2017 (n=364) [34], Lawes 2014 (n=155) [35]<br>Neu 2003 (n=2,121) [52]<br>Demir 2015 (n=395) [42]	Shaltout 2016 (n=679) [53]<br>Ahmed 2020 (n=982) [54]		OR 2.01 (1.05-4.14)[35]; OR 1.79 (1.01–3.18)[51]; OR 1.6 (1.1–2.4) [34];<br>19-23.8% vs. 30-55.1%:<br>- 23.8 vs. 28.9% [52]<br>- 19 vs. 30% [14]<br>- 41.7 vs. 55.1% [42]
Severe DKA (n=2,626)	Szypowska 2017 (n=2,100) [55], Aminzadeh 2019 (n=297) [51], Cutfield 2011 (n=229) [14]			OR 1.94 (1.32-2.84) [55]; 9.0 vs. 36.0% [14]
DKA during treatment (n=1,243)	Rewers 2002 (n=1,243) [56]			
Hypoglycaemia (n=4,837)			Bulsara 2004 (n=1,335) [57]<br>Craig 2002 (n=1,190) [58],<br>Craig 2007 (n=2,312) [33]	OR 1.44 (1.11–1.86) [57]; RR 1.13(1.02–1.26) [33]; IRR 1.49 (1.01-2.19) [58]
Hospital admissions (n=29,014)	Bohn 2018 (n=26,444) [59]<br>Cohn 1997 (n=1,676) [60]<br>Alaghehbandan 2006 (n=894) [61]			Hospitalisation rate 77.3 vs. 100.2/100,000 PY [61];<br>Multiple hospitalisations 18 vs. 26% [60]

DKA admissions (n=2,570)	Cohn 1997 (n=1,676) [60] Alaghehbandan 2006 (n=894) [61]			14.9 vs. 28.2/100,000 PY [61]; >1 DKA hospitalisation 14.2 vs. 23.5% [60]
Length of hospital stay (n=1,564)	Icks 2001 (n=373) [62] Aminzadeh 2019 (n=297) [51]		Alaghehbandan 2006 (n=894) [61]	8.7 ± 3.93 vs. 10.2± 5.08 days [51]
Nephropathy (n=96,293)	Lin 2016 (n=96,171) [63]		Di Bonito 2019 (n=122) [64]	33 vs. 57% [64] ; 3.04 vs. 3.86/100 patients [63]
Retinopathy (n=194)	Falck 1993 (n=194) [65]			16.7 vs. 36.4% [65] (age >13 years)
**Comorbidity**				
Thyroid disease/ antibodies (n=5,076)	Spaans 2017 (n=4,186) [66] Demir 2015 (n=395) [42] Holl 1999 (n=495) [67]	Menon 2001 (n=67) [68]		Rate ratio 3.07 (2.10-4.49) [66]
Coeliac disease biopsy proven (n=53,104)	Craig 2017 (n=52,721) [69] Valerio 2002 (n=383) [70]			2.7 vs. 4.3% [69]
tTGA (n=2,705)	Bybrant 2018 (n=2,705) [71]			4.2 vs. 6.6% [71]
Metabolic syndrome (n=1,662)	Barros 2017 (n=1,662) [72]			
Depression (n=744)	Glick 2018 (n=530) [73] Picozzi 2019 (n=214) [74]			
Eating disorder (n=178)	Cecilia-Costa 2019 (n=178) [75]	Troncone 2019 (n=108) [76]		Moderate 19% vs 34% or high 10% vs 20% ED behaviour levels [75]
Anxiety (n=187)	Al Hayek 2015 (n=187) [77]			
ADHD (n=3,668)			Kapellen 2016 (n=3,668) [78]	4.1 vs. 1.7% [78]
**Quality of life**				
Overall Qol (n=5,866)	Lawrence 2012 (n=2,602) [79] Albuhairan 2016 (n=315) [80] Chaplin 2009 (n=361) [81] Frøisland 2013 (n=937) [82] Hanberger 2009 (n=400) [83] Lukács 2016 (n=296) [84] Lukács 2018 (n=229) [85] Mozzillo 2017 (n=242) [86] Petersson 2015 (n=217) [87] Hassan 2017 (n=150) [88] Kalyva 2011 (n=117) [89]			DISABKIDS General HrQoL score 81 [41-100] vs. 79 [40-99] [81]; 78 ±13 vs. 71±15 [87]. PedsQL generic scales 79.74 ±11.16 vs. 75.71±12.96 [84], PedsQL diabetes module 74.11 ±10.47 vs. 70.26±13.04 [84]; 73.00 ±9.88 vs. 70.02 ±13.15 [85]. DAWN Youth QoL Tool 29.1 ±9.5 vs. 25.2 ± 7.3 [88]
Certain subdomains (n=2,403)	Hoey 2001 (n=2,101) [19], Graue 2003 (n=115) [90], Al Hayek 2015 (n=187) [77]			
Diabetes related distress (n=453)	Forsander 2017 (n=453) [38]			19 vs. 44% [38]
Fear of hypoglycaemia (n=453)	Forsander 2017 (n=453) [38]			

**ESM Table 3** Study outcomes for HbA_{1c} during follow-up				
**Study**	**Age**	**Mean HbA_{1c} male**	**Mean HbA_{1c} female**	**Comment**
Allen 1992 [41]	0-9	10.4%	10.9%	Measured as glycated haemoglobin (GHb) Average year 1&2
		(90.2 mmol/mol)	(95.6 mmol/mol)	
Gomes 2013 [26]	1-18	9.2 ± 2.4%	9.6 ± 2.5%	p = 0.004
		(77.0mmol/mol)	(81.4 mmol/mol)	
Hanberger 2014 [32]	0-18	7.7%	7.825%	Average all age categories
		(60.25 mmol/mol)	(61.75 mmol/mol)	
Hanberger 2018 [17]	11-16	61.3 mmol/mol	63.3 mmol/mol	
		(7.8%)	(7.9%)	
Hoey 2001 [19]	10-18	8.6%±1.6	9.0%±1.7	
		(70.5mmol/mol)	(74.9 mmol/mol)	
Mortensen 1988 [21]	0-18	77mmol/mol	80 mmol/mol	Average age categories
		(9.2%)	(9.45%)	
Samuelsson 2016 [18]	13-18	66±15 mmol/mol	69±15mmol/mol	
		(8.2%±1.4)	(8.5%±1.5)	
Maffeis 2018 [5]	2-18	8.06% ± 0.10%	8.20% ± 0.10%	p<0.0001 Adjusted for age and duration of diabetes
		(64.6 mmol/mol)	(66.1 mmol/mol)	
Schwab 2010 [7]	≤18	8.2 ±0.01	8.3±0.01	p<0.001
		(66.1 mmol/mol)	(67.2 mmol/mol)	
Forsander 2016 [38]	15-18	56 ±12.9 mmol/mol	62.4 ±15.4 mmol/mol	p< 0.0001
		(7.3%)	(7.9%)	

**ESM Table 4** Study outcomes for BMI-SDS during treatment				
**Study**	**Age**	**BMI-SDS male**	**BMI-SDS female**	**Comment**
Birkebaek 2018 [16]	<15	0.67	0.72	p = 0.03
Hanberger 2018 [17]	11-16	0.5	0.57	Average of countries
Maffeis 2018 [5]	2-18	0.40 ± 0.05	0.54 ± 0.05	p<0.0001 Adjusted for age and duration of diabetes
Manyanga 2016 [15]	2-18	0.52	0.69	
Łuczyński 2014 [11]	4-18	0.46	0.64	
Schwab 2010 [7]	≤18	0.41	0.60	
Dost 2008 [8]	5-18	0.42 ± 0.03	0.57 ± 0.03	
Samuelsson 2016 [18]	13-18	0.4 ± 1.0	0.7 ± 0.9	

ESM references

1. Turtinen M, Härkönen T, Parkkola A, Ilonen J, Knip M (2018) Sex as a determinant of type 1 diabetes at diagnosis. Pediatr Diabetes 19(7):1221–1228. https://doi.org/10.1111/pedi.12697

2. Dost A, Herbst A, Kintzel K, et al (2007) Shorter remission period in young versus older children with diabetes mellitus type 1. Exp Clin Endocrinol diabetes Off journal, Ger Soc Endocrinol [and] Ger Diabetes Assoc 115(1):33–37. https://doi.org/10.1055/s-2007-948214

3. Marino KR, Lundberg RL, Jasrotia A, et al (2017) A predictive model for lack of partial clinical remission in new-onset pediatric type 1 diabetes. PLoS One 12(5):e0176860. https://doi.org/10.1371/journal.pone.0176860

4. Ortqvist E, Falorni A, Scheynius A, Persson B, Lernmark A (1997) Age governs gender-dependent islet cell autoreactivity and predicts the clinical course in childhood IDDM. Acta Paediatr 86(11):1166–1171. https://doi.org/10.1111/j.1651-2227.1997.tb14837.x

5. Maffeis C, Birkebaek NH, Konstantinova M, et al (2018) Prevalence of underweight, overweight, and obesity in children and adolescents with type 1 diabetes: Data from the international SWEET registry. Pediatr Diabetes 19(7):1211–1220. https://doi.org/10.1111/pedi.12730

6. Plamper M, Gohlke B, Woelfle J, et al (2017) Interaction of Pubertal Development and Metabolic Control in Adolescents with Type 1 Diabetes Mellitus. J Diabetes Res 2017:8615769. https://doi.org/10.1155/2017/8615769

7. Schwab KO, Doerfer J, Marg W, Schober E, Holl RW (2010) Characterization of 33 488 children and adolescents with type 1 diabetes based on the gender-specific increase of cardiovascular risk factors. Pediatr Diabetes 11(5):357–363. https://doi.org/10.1111/j.1399-5448.2010.00665.x

8. Dost A, Klinkert C, Kapellen T, et al (2008) Arterial hypertension determined by ambulatory blood pressure profiles: contribution to microalbuminuria risk in a multicenter investigation in 2,105 children and adolescents with type 1 diabetes. Diabetes Care 31(4):720–725. https://doi.org/10.2337/dc07-0824

9. Łuczyński W, Szypowska A, Głowińska-Olszewska B, Bossowski A (2011) Overweight, obesity and features of metabolic syndrome in children with diabetes treated with insulin pump therapy. Eur J Pediatr 170(7):891–898. https://doi.org/10.1007/s00431-010-1372-7

10. Kapellen TM, Gausche R, Dost A, et al (2014) Children and adolescents with type 1 diabetes in Germany are more overweight than healthy controls: results comparing DPV database and CrescNet database. J Pediatr Endocrinol Metab 27(3–4):209–214. https://doi.org/10.1515/jpem-2013-0381

11. Łuczyński W, Szypowska A, Głowińska-Olszewska B, Szadkowska A, Bossowski A (2014) Disease associated clinical factors and FTO polymorphism: effect on body mass in children with type 1 diabetes mellitus. Pediatr Diabetes 15(5):363–371. https://doi.org/10.1111/pedi.12091

12. Islam ST, Abraham A, Donaghue KC, et al (2014) Plateau of adiposity in Australian children diagnosed with Type 1 diabetes: a 20-year study. Diabet Med 31(6):686–690. https://doi.org/10.1111/dme.12402

13. Kibirige M, Metcalf B, Renuka R, Wilkin TJ (2003) Testing the accelerator hypothesis: the relationship between body mass and age at diagnosis of type 1 diabetes. Diabetes Care 26(10):2865–2870. https://doi.org/10.2337/diacare.26.10.2865

14. Cutfield SW, Derraik JGB, Reed PW, Hofman PL, Jefferies C, Cutfield WS (2011) Early markers of glycaemic control in children with type 1 diabetes mellitus. PLoS One 6(9):e25251. https://doi.org/10.1371/journal.pone.0025251

15. Manyanga T, Sellers EA, Wicklow BA, Doupe M, Fransoo R (2016) Is the change in body mass index among children newly diagnosed with type 1 diabetes mellitus associated with obesity at transition from pediatric to adult care? Pediatr Diabetes 17(8):599–607. https://doi.org/10.1111/pedi.12344

16. Birkebaek NH, Kahlert J, Bjarnason R, et al (2018) Body mass index standard deviation score and obesity in children with type 1 diabetes in the Nordic countries. HbA(1c) and other predictors of increasing BMISDS. Pediatr Diabetes 19(7):1198–1205. https://doi.org/10.1111/pedi.12693

17. Hanberger L, Samuelsson U, Holl RW, Fröhlich-Reiterer E, Åkesson K, Hofer S (2018) Type 1 diabetes during adolescence: International comparison between Germany, Austria, and Sweden. Pediatr Diabetes 19(3):506–511. https://doi.org/10.1111/pedi.12591

18. Samuelsson U, Anderzén J, Gudbjörnsdottir S, Steineck I, Åkesson K, Hanberger L (2016) Teenage girls with type 1 diabetes have poorer metabolic control than boys and face more complications in early adulthood. J Diabetes Complications 30(5):917–922. https://doi.org/10.1016/j.jdiacomp.2016.02.007

19. Hoey H, Aanstoot HJ, Chiarelli F, et al (2001) Good metabolic control is associated with better quality of life in 2,101 adolescents with type 1 diabetes. Diabetes Care 24(11):1923–1928. https://doi.org/10.2337/diacare.24.11.1923

20. Dorchy H, Roggemans MP, Willems D (1997) Glycated hemoglobin and related factors in diabetic children and adolescents under 18 years of age: a Belgian experience. Diabetes Care 20(1):2–6. https://doi.org/10.2337/diacare.20.1.2

21. Mortensen HB, Hartling SG, Petersen KE (1988) A nation-wide cross-sectional study of glycosylated haemoglobin in Danish children with type 1 diabetes. Diabet Med 5(9):871–876. https://doi.org/10.1111/j.1464-5491.1988.tb01127.x

22. Moore JM, Snell-Bergeon JK (2019) Trajectories of hemoglobin A1c and body mass index z-score over four decades among 2 to 18 year olds with type 1 diabetes. Pediatr Diabetes 20(5):594–603. https://doi.org/10.1111/pedi.12862

23. Phelan H, Foster NC, Schwandt A, et al (2020) Longitudinal trajectories of BMI z-score: an international comparison of 11,513 Australian, American and German/Austrian/Luxembourgian youth with type 1 diabetes. Pediatr Obes 15(2):1–9. https://doi.org/10.1111/ijpo.12582

24. Minges KE, Whittemore R, Weinzimer SA, Irwin ML, Redeker NS, Grey M (2017) Correlates of overweight and obesity in 5529 adolescents with type 1 diabetes: The T1D Exchange Clinic Registry. Diabetes Res Clin Pract 126:68–78. https://doi.org/10.1016/j.diabres.2017.01.012

25. Mortensen HB, Hougaard P, Ibsen KK, Parving HH (1994) Relationship between blood pressure and urinary albumin excretion rate in young Danish type 1 diabetic patients: comparison to non-diabetic children. Danish Study Group of Diabetes in Childhood. Diabet Med 11(2):155–161. https://doi.org/10.1111/j.1464-5491.1994.tb02012.x

26. Gomes MB, de Mattos Matheus AS, Calliari LE, et al (2013) Economic status and clinical care in young type 1 diabetes patients: a nationwide multicenter study in Brazil. Acta Diabetol 50(5):743–752. https://doi.org/10.1007/s00592-012-0404-3

27. Kosteria I, Schwandt A, Davis E, Jali S, Prieto M, Rottembourg D (2019) Lipid profile is associated with treatment regimen in a large cohort of children and adolescents with Type 1 diabetes mellitus: a study from the international SWEET database. Diabet Med 36(10):1294–1303. https://doi.org/10.1111/dme.13963

28. Silverio RNC, de Aquino Lacerda EM, Fortins RF, et al (2019) Predictive factors of non-HDL cholesterol in children and adolescents with type 1 diabetes mellitius: A cross-sectional study. Diabetes Res Clin Pract 154:9–16. https://doi.org/10.1016/j.diabres.2019.06.005

29. Szypowska A, Groele L, Wysocka-Mincewicz M, et al (2018) Factors associated with preservation of C-peptide levels at the diagnosis of type 1 diabetes. J Diabetes Complications 32(6):570–574. https://doi.org/10.1016/j.jdiacomp.2018.03.009

30. Samuelsson U, Lindblad B, Carlsson A, et al (2013) Residual beta cell function at diagnosis of type 1 diabetes in children and adolescents varies with gender and season. Diabetes Metab Res Rev 29(1):85–89. https://doi.org/10.1002/dmrr.2365

31. Quinn M, Fleischman A, Rosner B, Nigrin DJ, Wolfsdorf JI (2006) Characteristics at diagnosis of type 1 diabetes in children younger than 6 years. J Pediatr 148(3):366–371. https://doi.org/10.1016/j.jpeds.2005.10.029

32. Hanberger L, Åkesson K, Samuelsson U (2014) Glycated haemoglobin variations in paediatric type 1 diabetes: the impact of season, gender and age. Acta Paediatr 103(4):398–403. https://doi.org/10.1111/apa.12530

33. Craig ME, Jones TW, Silink M, Ping YJ (2007) Diabetes care, glycemic control, and complications in children with type 1 diabetes from Asia and the Western Pacific Region. J Diabetes Complications 21(5):280–287. https://doi.org/10.1016/j.jdiacomp.2006.04.005

34. Khanolkar AR, Amin R, Taylor-Robinson D, et al (2017) Diabetic Ketoacidosis Severity at Diagnosis and Glycaemic Control in the First Year of Childhood Onset Type 1 Diabetes-A Longitudinal Cohort Study. Int J Environ Res Public Health 15(1):26. https://doi.org/10.3390/ijerph15010026

35. Lawes T, Franklin V, Farmer G (2014) HbA1c tracking and bio-psychosocial determinants of glycaemic control in children and adolescents with type 1 diabetes: retrospective cohort study and multilevel analysis. Pediatr Diabetes 15(5):372–383. https://doi.org/10.1111/pedi.12100

36. Rohan JM, Rausch JR, Pendley JS, et al (2014) Identification and prediction of group-based glycemic

control trajectories during the transition to adolescence. Heal Psychol Off J Div Heal Psychol Am Psychol Assoc 33(10):1143–1152. https://doi.org/10.1037/hea0000025

37. Springer D, Dziura J, Tamborlane W V, et al (2006) Optimal control of type 1 diabetes mellitus in youth receiving intensive treatment. J Pediatr 149(2):227–232. https://doi.org/10.1016/j.jpeds.2006.03.052

38. Forsander G, Bøgelund M, Haas J, Samuelsson U (2017) Adolescent life with diabetes-Gender matters for level of distress. Experiences from the national TODS study. Pediatr Diabetes 18(7):651–659. https://doi.org/10.1111/pedi.12478

39. McKnight JA, Wild SH, Lamb MJE, et al (2015) Glycaemic control of Type 1 diabetes in clinical practice early in the 21st century: an international comparison. Diabet Med 32(8):1036–1050. https://doi.org/10.1111/dme.12676

40. Yazar A, Akın F, Akça ÖF, et al (2019) The effect of attention deficit/hyperactivity disorder and other psychiatric disorders on the treatment of pediatric diabetes mellitus. Pediatr Diabetes 20(3):345–352. https://doi.org/10.1111/pedi.12819

41. Allen C, Zaccaro DJ, Palta M, Klein R, Duck SC, D'Alessio DJ (1992) Glycemic control in early IDDM. The Wisconsin Diabetes Registry. Diabetes Care 15(8):980–987. https://doi.org/10.2337/diacare.15.8.980

42. Demir F, Günöz H, Saka N, et al (2015) Epidemiologic Features of Type 1 Diabetic Patients between 0 and 18 Years of Age in İstanbul City. J Clin Res Pediatr Endocrinol 7(1):49–56. https://doi.org/10.4274/jcrpe.1694

43. Mortensen HB, Robertson KJ, Aanstoot HJ, et al (1998) Insulin management and metabolic control of type 1 diabetes mellitus in childhood and adolescence in 18 countries. Hvidøre Study Group on Childhood Diabetes. Diabet Med 15(9):752–759. https://doi.org/10.1002/(SICI)1096-9136(199809)15:9<752::AID-DIA678>3.0.CO;2-W

44. Wiegand S, Raile K, Reinehr T, et al (2008) Daily insulin requirement of children and adolescents with type 1 diabetes: effect of age, gender, body mass index and mode of therapy. Eur J Endocrinol 158(4):543–549. https://doi.org/10.1530/EJE-07-0904

45. Komulainen J, Akerblom HK, Lounamaa R, Knip M (1998) Prepubertal girls with insulin-dependent diabetes mellitus have higher exogenous insulin requirement than boys. Childhood Diabetes in Finland Study Group. Eur J Pediatr 157(9):708–711. https://doi.org/10.1007/s004310050919

46. Herbst A, Roth CL, Dost AG, Fimmers R, Holl RW (2005) Rate of hypoglycaemia and insulin dosage in children during the initial therapy of type 1 diabetes mellitus. Eur J Pediatr 164(10):633–638. https://doi.org/10.1007/s00431-005-1723-y

47. Rasmussen VF, Vestergaard ET, Schwandt A, et al (2019) Proportion of Basal to Total Insulin Dose Is Associated with Metabolic Control, Body Mass Index, and Treatment Modality in Children with Type 1 Diabetes-A Cross-Sectional Study with Data from the International SWEET Registry. J Pediatr 215:216-222.e1. https://doi.org/10.1016/j.jpeds.2019.06.002

48. Bächle C, Icks A, Straßburger K, et al (2013) Direct diabetes-related costs in young patients with early-onset, long-lasting type 1 diabetes. PLoS One 8(8):e70567. https://doi.org/10.1371/journal.pone.0070567

49. Sherr JL, Hermann JM, Campbell F, et al (2016) Use of insulin pump therapy in children and adolescents with type 1 diabetes and its impact on metabolic control: comparison of results from three large, transatlantic paediatric registries. Diabetologia 59(1):87–91. https://doi.org/10.1007/s00125-015-3790-6

50. van den Boom L, Karges B, Auzanneau M, et al (2019) Temporal Trends and Contemporary Use of Insulin Pump Therapy and Glucose Monitoring Among Children, Adolescents, and Adults With Type 1 Diabetes Between 1995 and 2017. Diabetes Care 42(11):2050–2056. https://doi.org/10.2337/dc19-0345

51. Aminzadeh M, Navidi N, Valavi E, Aletayeb SMH (2019) Childhood onset type 1 diabetes at a tertiary hospital in south-western Iran during 2000-2015: Rapid increase in admissions and high prevalence of DKA at diagnosis. Prim Care Diabetes 13(1):43–48. https://doi.org/10.1016/j.pcd.2018.07.013

52. Neu A, Willasch A, Ehehalt S, Hub R, Ranke MB (2003) Ketoacidosis at onset of type 1 diabetes mellitus in children--frequency and clinical presentation. Pediatr Diabetes 4(2):77–81. https://doi.org/10.1034/j.1399-5448.2003.00007.x

53. Shaltout AA, Channanath AM, Thanaraj TA, et al (2016) Ketoacidosis at first presentation of type 1 diabetes mellitus among children: A study from Kuwait. Sci Rep 6:1–9. https://doi.org/10.1038/srep27519

54. Ahmed AM, Khabour OF, Ahmed SM, Alebaid IA, Ibrahim AM (2020) Frequency and severity of ketoacidosis at diagnosis among childhood type 1 diabetes in khartoum state, sudan. Afr Health Sci

20(2):841–848. https://doi.org/10.4314/ahs.v20i2.38

55. Szypowska A, Dżygało K, Wysocka-Mincewicz M, et al (2017) High incidence of diabetic ketoacidosis at diagnosis of type 1 diabetes among Polish children aged 10-12 and under 5 years of age: A multicenter study. Pediatr Diabetes 18(8):722–728. https://doi.org/10.1111/pedi.12446

56. Rewers A, Chase HP, Mackenzie T, et al (2002) Predictors of acute complications in children with type 1 diabetes. JAMA 287(19):2511–2518. https://doi.org/10.1001/jama.287.19.2511

57. Bulsara MK, Holman CDJ, Davis EA, Jones TW (2004) The impact of a decade of changing treatment on rates of severe hypoglycemia in a population-based cohort of children with type 1 diabetes. Diabetes Care 27(10):2293–2298. https://doi.org/10.2337/diacare.27.10.2293

58. Craig ME, Handelsman P, Donaghue KC, et al (2002) Predictors of glycaemic control and hypoglycaemia in children and adolescents with type 1 diabetes from NSW and the ACT. Med J Aust 177(5):235–238

59. Bohn B, Schwandt A, Ihle P, et al (2018) Hospital admission in children and adolescents with or without type 1 diabetes from Germany: An analysis of statutory health insurance data on 12 million subjects. Pediatr Diabetes 19(4):721–726. https://doi.org/10.1111/pedi.12621

60. Cohn BA, Cirillo PM, Wingard DL, Austin DF, Roffers SD (1997) Gender differences in hospitalizations for IDDM among adolescents in California, 1991. Implications for prevention. Diabetes Care 20(11):1677–1682. https://doi.org/10.2337/diacare.20.11.1677

61. Alaghehbandan R, Collins KD, Newhook LA, MacDonald D (2006) Childhood type 1 diabetes mellitus in Newfoundland and Labrador, Canada. Diabetes Res Clin Pract 74(1):82–89. https://doi.org/10.1016/j.diabres.2006.03.001

62. Icks A, Rosenbauer J, Haastert B, Giani G (2001) Hospitalization among diabetic children and adolescents and non-diabetic control subjects: a prospective population-based study. Diabetologia 44(Suppl 3):B87-92. https://doi.org/10.1007/pl00002960

63. Li L, Jick S, Breitenstein S, Michel A (2016) Prevalence of Diabetes and Diabetic Nephropathy in a Large U.S. Commercially Insured Pediatric Population, 2002-2013. Diabetes Care 39(2):278–284. https://doi.org/10.2337/dc15-1710

64. Di Bonito P, Mozzillo E, Esposito M, et al (2019) Non-albuminuric reduced eGFR phenotype in children and adolescents with type 1 diabetes. Diabetes Res Clin Pract 155:107781. https://doi.org/10.1016/j.diabres.2019.07.005

65. Falck AA, Käär ML, Laatikainen LT (1993) Prevalence and risk factors of retinopathy in children with diabetes. A population-based study on Finnish children. Acta Ophthalmol 71(6):801–809. https://doi.org/10.1111/j.1755-3768.1993.tb08604.x

66. Spaans E, Schroor E, Groenier K, Bilo H, Kleefstra N, Brand P (2017) Thyroid Disease and Type 1 Diabetes in Dutch Children: A Nationwide Study (Young Dudes-3). J Pediatr 187:189-193.e1. https://doi.org/10.1016/j.jpeds.2017.05.016

67. Holl RW, Bohm B, Loos U, Grabert M, Heinze E, Homoki J (1999) Thyroid autoimmunity in children and adolescents with type 1 diabetes mellitus. Effect of age, gender and HLA type. Horm Res 52(3):113–118. https://doi.org/10.1159/000023446

68. Menon PSN, Vaidyanathan B, Kaur M (2001) Autoimmune thyroid disease in Indian children with type 1 diabetes mellitus. J Pediatr Endocrinol Metab 14(3):279–286. https://doi.org/10.1515/JPEM.2001.14.3.279

69. Craig ME, Prinz N, Boyle CT, et al (2017) Prevalence of Celiac Disease in 52,721 Youth With Type 1 Diabetes: International Comparison Across Three Continents. Diabetes Care 40(8):1034–1040. https://doi.org/10.2337/dc16-2508

70. Valerio G, Maiuri L, Troncone R, et al (2002) Severe clinical onset of diabetes and increased prevalence of other autoimmune diseases in children with coeliac disease diagnosed before diabetes mellitus. Diabetologia 45(12):1719–1722. https://doi.org/10.1007/s00125-002-0923-5

71. Cerqueiro Bybrant M, Grahnquist L, Örtqvist E, et al (2018) Tissue transglutaminase autoantibodies in children with newly diagnosed type 1 diabetes are related to human leukocyte antigen but not to islet autoantibodies: A Swedish nationwide prospective population-based cohort study. Autoimmunity 51(5):221–227. https://doi.org/10.1080/08916934.2018.1494160

72. Barros BSV, Santos DC, Pizarro MH, del Melo LGN, Gomes MB (2017) Type 1 Diabetes and Non-Alcoholic Fatty Liver Disease: When Should We Be Concerned? A Nationwide Study in Brazil. Nutrients

9(8):878. https://doi.org/10.3390/nu9080878

73. Glick BA, Hong KMC, Obrynba K, Kamboj MK, Hoffman RP (2018) Identifying depressive symptoms among diabetes type and the impact on hemoglobin A1c. J Pediatr Endocrinol Metab 31(1):39–44. https://doi.org/10.1515/jpem-2017-0241

74. Picozzi A, DeLuca F (2019) Depression and glycemic control in adolescent diabetics: evaluating possible association between depression and hemoglobin A1c. Public Health 170:32–37. https://doi.org/10.1016/j.puhe.2019.02.005

75. Cecilia-Costa R, Volkening LK, Laffel LM (2019) Factors associated with disordered eating behaviours in adolescents with Type 1 diabetes. Diabet Med 36(8):1020–1027. https://doi.org/10.1111/dme.13890

76. Troncone A, Cascella C, Chianese A, et al (2019) Parental assessment of disordered eating behaviors in their children with type 1 diabetes: A controlled study. J Psychosom Res 119(February):20–25. https://doi.org/10.1016/j.jpsychores.2019.02.003

77. Al Hayek AA, Robert AA, Braham RB, Issa BA, Al Sabaan FS (2015) Predictive Risk Factors for Fear of Hypoglycemia and Anxiety-Related Emotional Disorders among Adolescents with Type 1 Diabetes. Med Princ Pract Int J Kuwait Univ Heal Sci Cent 24(3):222–230. https://doi.org/10.1159/000375306

78. Kapellen TM, Reimann R, Kiess W, Kostev K (2016) Prevalence of medically treated children with ADHD and type 1 diabetes in Germany - Analysis of two representative databases. J Pediatr Endocrinol Metab 29(11):1293–1297. https://doi.org/10.1515/jpem-2016-0171

79. Lawrence JM, Yi-Frazier JP, Black MH, et al (2012) Demographic and clinical correlates of diabetes-related quality of life among youth with type 1 diabetes. J Pediatr 161(2):201–7.e2. https://doi.org/10.1016/j.jpeds.2012.01.016

80. AlBuhairan F, Nasim M, Al Otaibi A, Shaheen NA, Al Jaser S, Al Alwan I (2016) Health related quality of life and family impact of type 1 diabetes among adolescents in Saudi Arabia. Diabetes Res Clin Pract 114:173–179. https://doi.org/10.1016/j.diabres.2016.01.001

81. Chaplin JE, Hanas R, Lind A, Tollig H, Wramner N, Lindblad B (2009) Assessment of childhood diabetes-related quality-of-life in West Sweden. Acta Paediatr 98(2):361–366. https://doi.org/10.1111/j.1651-2227.2008.01066.x

82. Frøisland DH, Graue M, Markestad T, Skrivarhaug T, Wentzel-Larsen T, Dahl-Jørgensen K (2013) Health-related quality of life among Norwegian children and adolescents with type 1 diabetes on intensive insulin treatment: a population-based study. Acta Paediatr 102(9):889–895. https://doi.org/10.1111/apa.12312

83. Hanberger L, Ludvigsson J, Nordfeldt S (2009) Health-related quality of life in intensively treated young patients with type 1 diabetes. Pediatr Diabetes 10(6):374–381. https://doi.org/10.1111/j.1399-5448.2008.00496.x

84. Lukács A, Sasvári P, Török A, Barkai L (2016) Generic and disease-specific quality of life in adolescents with type 1 diabetes: comparison to age-matched healthy peers. J Pediatr Endocrinol Metab 29(7):769–775. https://doi.org/10.1515/jpem-2015-0397

85. Lukács A, Mayer K, Sasvári P, Barkai L (2018) Health-related quality of life of adolescents with type 1 diabetes in the context of resilience. Pediatr Diabetes 19(8):1481–1486. https://doi.org/10.1111/pedi.12769

86. Mozzillo E, Zito E, Maffeis C, et al (2017) Unhealthy lifestyle habits and diabetes-specific health-related quality of life in youths with type 1 diabetes. Acta Diabetol 54(12):1073–1080. https://doi.org/10.1007/s00592-017-1051-5

87. Petersson C, Huus K, Samuelsson U, Hanberger L, Akesson K (2015) Use of the national quality registry to monitor health-related quality of life of children with type 1 diabetes: a pilot study. J child Heal care Prof Work with Child Hosp community 19(1):30–42. https://doi.org/10.1177/1367493513496674

88. Hassan M, Musa N, Abdel Hai R, Fathy A, Ibrahim A (2017) Assessment of health-related quality of life in Egyptian adolescents with type 1 diabetes: DEMPU survey. J Pediatr Endocrinol Metab 30(3):277–283. https://doi.org/10.1515/jpem-2016-0147

89. Kalyva E, Malakonaki E, Eiser C, Mamoulakis D (2011) Health-related quality of life (HRQoL) of children with type 1 diabetes mellitus (T1DM): self and parental perceptions. Pediatr Diabetes 12(1):34–40. https://doi.org/10.1111/j.1399-5448.2010.00653.x

90. Graue M, Wentzel-Larsen T, Hanestad BR, Båtsvik B, Søvik O (2003) Measuring self-reported, health-

related, quality of life in adolescents with type 1 diabetes using both generic and disease-specific instruments. Acta Paediatr 92(10):1190–1196
